# Co-evolution of Cognitive and Health Trajectories Among U.S. Older Adults with Cognitive Impairment

**DOI:** 10.1093/geroni/igaf122.2900

**Published:** 2025-12-31

**Authors:** Yifan Lou, Emma Zang, Yanjun Wu

**Affiliations:** Virginia Commonwealth University, Richmond, Virginia, United States; Yale University, New Haven, Connecticut, United States; Cleveland Clinic, Cleveland, Ohio, United States

## Abstract

Few studies have explored how multiple health aspects co-evolve over time among persons with cognitive impairment. This study examines the interplays of health status and cognitive trajectories in persons with cognitive impairment. We examine how health status trajectories predict cognitive trajectories, focusing on conditional probabilities to highlight how broader health dynamics shape cognition through shared biological and behavioral pathways. We analyzed data from 2,569 persons with cognitive impairment (PCIs) in the 2011–2021 National Health and Aging Trends Survey. Bayesian dual trajectory models identified distinct health status and cognitive trajectories. We examined their sociodemographic and health correlates. Conditional probabilities of cognitive group membership based on health status group membership were also estimated. We identified five distinct trajectory groups each for health status and cognition, ranging from high-stable to low-declining patterns. The most common health trajectory is “Medium start, moderate decline” (28.8%), followed by “High start, stable”. For cognition, the largest group of people fall into the “Medium start, slight decline” (29.7%) category, with “Medium-high start, stable” next (27.6%). Respondents with the most favorable health status trajectories were more likely to have the best cognitive outcomes, while those with sharp declines in health status experienced worse cognitive outcomes, despite starting at higher baseline health levels. To conclude, we find that stable health trajectories are strongly linked to better cognitive outcomes, while sharp health declines predict poorer cognition. These findings emphasize the need for integrated interventions to support physical and cognitive health in older adults.

